# Development and application of a quantitative polymerase chain reaction assay for the detection and genotyping of bovine leukemia virus in cattle from Kazakhstan

**DOI:** 10.14202/vetworld.2025.2320-2331

**Published:** 2025-08-14

**Authors:** Alexandr Ostrovskii, Alexandr Shevtsov, Marat Kuibagarov, Dinara Kamalova, Ayan Dauletov, Aralbek Rsaliyev, Yergali Abduraimov, Kassym Mukanov

**Affiliations:** 1 National Center for Biotechnology, 01000, Astana, Kazakhstan; 2 National Holding “QazBioPharm”, Astana, Kazakhstan

**Keywords:** bovine leukemia virus epidemiology, bovine leukemia virus, cattle, *env* gene, genotyping, Kazakhstan, molecular diagnostics, proviral DNA, quantitative polymerase chain reaction, *β-actin*

## Abstract

**Background and Aim:**

Bovine leukemia virus (BLV) is a globally distributed retrovirus that causes enzootic bovine leu-kosis, a chronic infection associated with significant economic losses in cattle. Conventional serological diagnostic tools such as agar gel immunodiffusion and enzyme-linked immunosorbent assay detect anti-BLV antibodies but cannot identify proviral DNA, especially in early infections or in calves with maternal antibodies. This study aimed to develop a sensitive and specific duplex quantitative polymerase chain reaction (qPCR) assay targeting the *env* gene of BLV with *β-actin* as an internal control and apply it for molecular surveillance and genotyping of BLV in cattle from six regions of Kazakhstan.

**Materials and Methods:**

A total of 1,680 bovine DNA samples from cattle aged over 3 years were collected from six administrative regions of Kazakhstan. A duplex qPCR assay was developed using primers targeting a conserved region of the BLV *env* gene and bovine *β-actin*. Sensitivity was assessed using plasmid and genomic DNA dilutions, and specificity was tested against existing WOAH-recommended and commercial polymerase chain reaction (PCR) protocols. Positive samples with cycle threshold <28 were subjected to nested PCR and Sanger sequencing for genotyping. Phylogenetic analysis was conducted using the maximum likelihood method.

**Results:**

The developed qPCR assay demonstrated a sensitivity of 20 plasmid copies for the *env* gene and 6 genomic equivalents for *β-actin* per reaction, with high specificity comparable to international standards. The overall BLV infection rate was 38.9%, ranging from 13% in Pavlodar to 60.5% in East Kazakhstan. Among 149 sequenced positive samples, four genotypes (G1, G4, G7, and G8) were identified. Genotype G4 was predominant, comprising 79.2% of sequences and present in all six regions.

**Conclusion:**

The duplex qPCR assay is a robust, sensitive, and cost-effective diagnostic tool for detecting BLV provirus, including in animals with maternal antibodies or early-stage infections. The regional genotypic distribution underscores the need for tailored control strategies. This molecular surveillance provides essential baseline data for national BLV eradication programs and contributes to global BLV epidemiological mapping.

## INTRODUCTION

Enzootic bovine leukosis (EBL) is the most prevalent viral neoplastic disease in cattle, causing significant economic losses due to reduced milk pro-duction, increased mortality, impaired reproductive performance, and the compulsory culling of infected animals [1–3]. The disease is caused by the bovine leukemia virus (BLV), an RNA retrovirus belonging to the *Retroviridae* family. In the absence of effective vaccines or antiviral therapies, EBL control primarily relies on regular disinfection and the identification and removal of infected animals. Preventive strategies include the exclusion of infected animals from herds and the enforcement of strict biosecurity and hygiene practices [4–6]. Both serological and molecular met-hods are employed for EBL diagnosis. Common serolo-gical tools used for BLV detection include agar gel immunodiffusion (AGID), passive hemagglutination assay, enzyme-linked immunosorbent assay (ELISA), and radioimmunoassay [7–10]. The World Organization for Animal Health (WOAH) recommends the use of AGID and ELISA in eradication programs due to their cost-effectiveness and suitability for large-scale screening [2].

However, serological assays may be insufficient in specific diagnostic contexts. These include testing calves with maternal antibodies, distinguishing between sporadic and enzootic lymphomas, examining tumor tissues post-slaughter, identifying infected carriers before seroconversion, confirming inconclusive ELISA res-ults, and screening animals used in biopharmaceutical production [11, 12]. In such cases, various forms of polymerase chain reaction (PCR) are widely employed to directly detect BLV proviral DNA in blood, milk, or tumor tissue samples. The first attempt to detect BLV proviral DNA was reported in 1990, but the method failed to identify the provirus in 72 seropositive animals [13]. To improve detection sensitivity and specificity, nested PCR protocols were subsequently developed [14–17]. Despite their high diagnostic performance, nested PCR methods are impractical for routine testing due to their complexity and the risk of cross-contamination during the transfer of samples between reaction steps [18]. The advent of real-time quantitative PCR (qPCR) addressed these limitations by automating the detection process, minimizing contamination, and significantly reducing diagnostic turnaround time [19]. Today, qPCR is widely used in veterinary laboratories for the detection of BLV [20–22], and its use is recommended by the WOAH Terrestrial Manual for testing individual animals during transport and for differentiating between sporadic and enzootic lymphoma cases [16].

In Kazakhstan, the average BLV infection rate is approximately 5.7%, though considerable regional and farm-level variation has been reported [23]. While AGID remains the standard diagnostic method for EBL screening in Kazakhstan, PCR is increasingly utilized for confirmatory diagnosis and for monitoring young animals during movement. To date, information reg-arding the genetic diversity of BLV genotypes circulating in Kazakhstan has been limited to studies from four reg-ions [23].

Although BLV is known to be endemic in Kazak-hstan, the current diagnostic surveillance is largely reli-ant on serological testing, which has limited sensitivity during early infection and in animals with maternal antibodies. While PCR-based methods offer greater diagnostic accuracy, their implementation remains inconsistent, and genotyping data are scarce and geographically limited. Previous molecular studies have only characterized BLV genotypes in four administrative regions, providing an incomplete picture of viral diver-sity across the country. Moreover, no standardized qPCR assay with an internal control has been systematically validated for large-scale field application in Kazakhstan. This hinders early detection, epidemiological mapping, and evidence-based eradication strategies.

This study aimed to develop and validate a duplex qPCR assay for detecting the BLV provirus by targeting a conserved region of the *env* gene, using *β-actin* as an internal control. The validated assay was then app-lied to a large cohort of cattle across six regions of Kazakhstan to assess the regional prevalence of BLV and to characterize the genetic diversity of circulating genotypes through phylogenetic analysis. The findings are intended to support national BLV control programs by providing a robust molecular diagnostic tool and updated epidemiological insights.

## MATERIALS AND METHODS

### Ethical approval

Ethical approval for sample collection was obtai-ned from Ethics Committee of the National Center for Biotechnology as part of a previous study by Kadyrova *et al*. [24] (ethical approval No. 2 dated April 04, 2022), which investigated the prevalence and species dive-rsity of *Anaplasma* spp. in cattle across all 17 regions of Kazakhstan.

### Study period and location

The study was conducted between September 2023 and July 2025 across six administrative regions of Kazakhstan: Aktobe, Akmola, East Kazakhstan, Pavlodar, North Kazakhstan, and Kostanay.

### Sample collection

A total of 1,680 DNA samples were collected from cattle aged over 3 years, reared on private subsidiary farms and grazed in communal herds across six regions of Kazakhstan: 190 samples from Aktobe, 200 each from Akmola and East Kazakhstan, 300 from Pavlodar, 362 from North Kazakhstan, and 428 from Kostanay. DNA was extracted from whole blood samples, and the procedures for both sample collection and DNA isolation are detailed in Kadyrova *et al*. [24].

### Primer design

Primers were designed based on 242 full-length BLV genome sequences retrieved from the Nati-onal Center for Biotechnology Information (NCBI) database (accessed November 27, 2023). Multiple sequence alignment was conducted using BioEdit ver-sion 7.2.5 [25]. Primers and a TaqMan fluorescent probe (AllGene, Almaty, Kazakhstan) were designed to target conserved regions of the *env* gene, selected for its relevance to viral infectivity and genotype coverage. For internal control, *β-actin* primers were developed by aligning coding sequences from several *Bovidae* species: *Bos taurus*, *Bos indicus*, *Bos javanicus*, *Bos mutus*, *Bubalus kerbau*, *Bubalus bubalis*, and *Bison bison*. A conserved exon region was selected due to its reduced genetic variability compared to introns [26]. Primer characteristics were assessed using Lasergene Primer Select 6.1 (DNASTAR Inc., Madison, WI, USA) and Primer-Basic Local Alignment Search Tool (Primer-BLAST; National Center for Biotechnology Information, Bethesda, MD, USA), and specificity was validated using the NCBI “nt” and “RefSeq representative genomes” databases [27].

### qPCR assay setup

The qPCR assay was conducted in a 35 μL reaction volume, comprising 17.5 μL of BioMaster UDG-HS-qPCR-2× master mix (Biolabmix, Russia), which contains uracil-DNA glycosylase (UDG) to prevent carryover contamination, primers and probes (Table 1), 7 μL of DNA template, and UltraPure™ DNase/RNase-Free distilled water (Invitrogen, USA). Nuclease-free distilled water served as a negative control. Amplification and real-time fluorescence detection were carried out using the QuantStudio 5 real-time PCR system (Applied Biosystems, Thermo Fisher Scientific, USA). The thermal cycling protocol included: UDG decontamination at 50°C for 2 min, initial denaturation at 95°C for 5 min, followed by 45 cycles–10 without fluorescence acquisition (95°C for 15 s, 60°C for 1 min), and 35 with acquisition during the 60°C annealing/extension step using FAM (for *env*) and VIC/HEX/JOE (for *β-actin*) fluorescence channels.

**Table 1:** Primer and TaqMan probe sets used in real-time PCR for detection of BLV provirus and bovine *β-actin*.

Primer/probe name	Sequencing 5’–3’	Length	Concentration in the PCR (nM)	Target
BLV_*env*_5461_F	GCCTTCCCAGACTGYGCYATATG	139 bp	500	*env* gene BLV (glycoprotein gp51.)
BLV_*env*_5600_R	AGGACGTGTTGACCCAGAAGAT		500	
BLV_*env*_probe 5494	TTCCCCTCCCTGGGCTCCCGA		300	
Betaactin_F_34-52	ACAGGAAGTCCTTTGCCTT	101 bp	500	*β-actin*
Betaactin_R_116-134	CACAAAAGCGATCACCTCC		400	
Betaactin_94-111_Probe	TCCTCGCCCGAGTCCACA		300	

PCR=Polymerase chain reaction, BLV=Bovine leukemia virus, bp=Base pair

### Sensitivity assessment

To evaluate assay sensitivity, a BLV *env* gene fragment was cloned into the pGEM^®^-T Easy vector (Promega, USA), transformed into *Escherichia coli* DH5α, and selected through blue–white screening on lysogeny broth agar plates with ampicillin, X-Gal, and isopropyl β-D-1-thiogalactopyranoside [28]. Positive colonies were cultured overnight and plasmid DNA was extracted using the Wizard Plus SV Miniprep Kit (Promega, Madison, WI, USA). Sanger sequencing confirmed insert identity. Plasmid copy numbers were quantified using a Qubit 2.0 (Invitrogen, Thermo Fisher Scientific, Carlsbad, CA, USA) fluorometer with the Qubit dsDNA HS Assay Kit (Invitrogen) and estimated using an online calculator (accessed October 12, 2024). Serial 4-fold dilutions ranged from 5,242,880 to 1.25 copies per reaction.

For *β-actin*, serial dilutions of bovine genomic DNA were quantified using the Qubit dsDNA BR Assay Kit (Invitrogen). Genome equivalents (GE) were calculated using Bos taurus genome size (~2.77 × 10^9^ base pair [bp]; NCBI accession: GCF_002263795.3), yielding dilutions from 1175.6 to 0.00028 ng/reaction (393,216–0.093 GE).

### Specificity assessment

Specificity was evaluated using 62 DNA samples from a commercial dairy herd with a high risk of BLV exposure. Samples were tested using the developed qPCR assay, a WOAH-recommended protocol [16], and a commercial kit (“PCR-Leukemia-Cattle Factor,” VetFactor, Russia). The WOAH protocol used a 25 μL reaction with 12.5 μL BioMaster mix, 0.4 μM primers (MRBLVL: 5’-CCTCAATTCCCTTTAAACTA-3’; MRBLVR: 5’-GT ACCGGGAAGACTGGATTA-3’), 0.2 μM probe (MRB LV_probe: 5’-6FAM-GAACGCCTCCAGGCCCTTCA-BHQ1-3’), and 7 μL DNA. Cycling conditions included 50°C for 2 min (UDG), 95°C for 5 min, and 50 cycles of 94°C for 1 min and 60°C for 1 min with FAM detection. All assays were run on the QuantStudio 5 Real-Time PCR System (Applied Biosystems, Thermo Fisher Scientific, USA).

### Statistical analysis

Because all three PCR assays were applied to the same sample set, paired Student’s *t*-tests were used to compare threshold cycle (Ct) values between methods. A p < 0.001 was considered statistically significant. Anal-yses were performed using the online GraphPad t-test calculator (https://www.graphpad.com/quickcalcs/ttes t1/?format=c).

### Identification of BLV-infected animals and genotyping

BLV-positive animals were identified via the developed qPCR assay. For genotyping, nested PCR tar-geting the *env* gene was employed. The first-round PCR used Moratorio *et al*.’s [29] primers (903 bp product): Forward 5’-ATGCCYAAAGAACGACGG-3’; rev-erse 5′-CGA CGGGACTAGGTCTGACCC-3′. In the second round, either a 594 bp (*env* 5032 and *env* 5608) or a 444 bp fragment (*env* 5099 and *env* 5521) was amp-lified [30]. Each 25 μL PCR reaction included 200 nM primers, 12.5 μL BioMaster HS-Taq PCR-Sp (2×) (Biolabmix, Russia), and 5 μL of genomic DNA (first round) or 3 μL of first-round product (second round). Conditions included 95°C for 5 min; 30 cycles of 95°C for 30 s, 55°C–70°C for 1 min (depending on amplicon), and 72°C for 1 min; with a final extension at 72°C for 5 min.

For samples failing initial amplification, an alternative nested PCR used BLV_4953_f and BLV_5580_R (first round), and BLV_4953_f and *env* 5521 (second round), under conditions of 94°C for 5 min; 40 cycles of 95°C for 30 s, 63°C for 40 s, 72°C for 60 s; and final extension at 72°C for 4 min. Ampl-icons were resolved on 1.5% agarose gels, visualized under UV light, and purified using magnetic beads according to the method described by Berdimuratova *et al*. [31].

### Phylogenetic analysis

Sanger sequencing was conducted using the BigDye Terminator v3.1 Cycle Sequencing Kit (Thermo Fisher Scientific, Vilnius, Lithuania) and analyzed on a 3730 × l Genetic Analyzer (Applied Biosystems, USA). Reads were assembled using SeqMan 6.1 (DNASTAR) [32]. Multiple sequence alignments were performed using Clust-alW in MEGA 12 (v12.0.11). Phylogenetic trees were constructed using the maximum likelihood method based on the Kimura 2-parameter model with gamma distribution, and node support was assessed via 1000 bootstrap replicates. Visualization was completed in MEGA 12 [33].

## RESULTS

### Primer development for BLV provirus and *β-actin* detection

The developed duplex PCR assay utilizes two sets of primers and probes: One specific to the BLV *env* gene and the other targeting bovine *β-actin* as an internal control. This dual-target design improves assay reliability by minimizing pipetting errors and reagent variability while providing a quality control for amplification efficiency. To optimize performance, primer design was guided by the selection of highly conserved target regions, integration of hot-start PCR protocols, and precise tuning of reagent concentrations. Detailed concentrations are listed in Table 1.

The *env* primers amplify a 139 bp region within the gene, designed to be complementary at the 3′ end across all 12 recognized BLV genotypes and full-genome sequences available in the NCBI database as of November 23, 2023. Across the full sequence, primer mismatches did not exceed one nucleotide. Degenerate bases were introduced at up to two polym-orphic sites per primer to accommodate viral diversity. The TaqMan probe sequence demonstrated complete complementarity at its 5′ end across all reference sequences, with only a single mismatch across its length.

Primers and probes for the *β-actin* gene were designed to target conserved exon regions across seven *Bovidae* species. BLAST analysis confirmed specific binding to target regions without off-target alignments. Secondary structure prediction showed no potential for hairpins, self-dimers, or other configurations that could interfere with amplification.

### Validation of sensitivity and specificity

#### Analytical sensitivity

**Figure 1:**
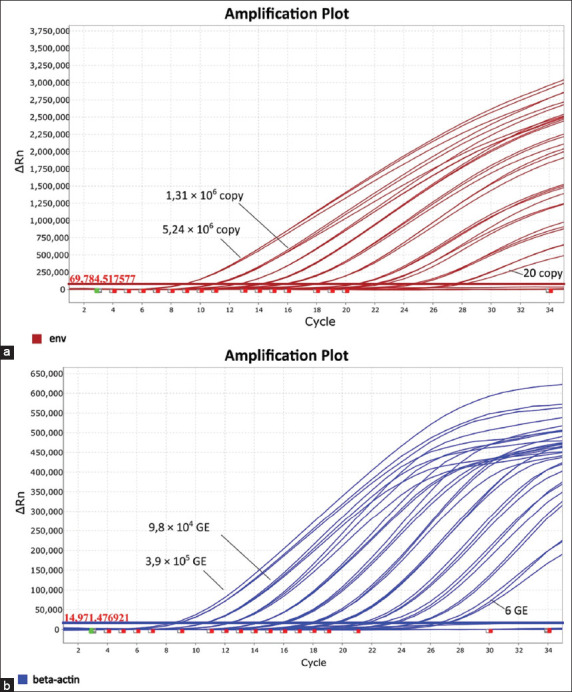
Sensitivity testing of quantitative polymerase chain reaction (qPCR) for detection of the bovine leukemia virus (BLV) *env* gene and *β-actin* in cattle. qPCR was performed using 4-fold serial dilutions. The dilution range for the plasmid was from 5,242,880 to 1.25 copies per reaction, and for the *β-actin* gene, from 393,216 to 0.093 genomic equivalents. (a) qPCR results for *env* BLV gene detection using plasmid DNA. (b) qPCR results for the detection of the *β-actin* gene using genomic DNA from cattle. Each sample was tested in triplicate. The red/blue horizontal line represents the fluorescence threshold.

The developed qPCR assay demonstrated a sensitivity of 20 plasmid copies per reaction for BLV *env* detection. No amplification signal was observed below this threshold. The internal control targeting *β-actin* had a sensitivity of 0.017 ng of genomic DNA, corresp-onding to approximately six genomic equivalents (GE). As shown in Figure 1, detection of the *env* gene ranged from 5.2 × 10^6^ to 20 copies per reaction, while *β-actin* detection ranged from 3.9 × 10^5^ to 6 GE.

**Figure 2:**
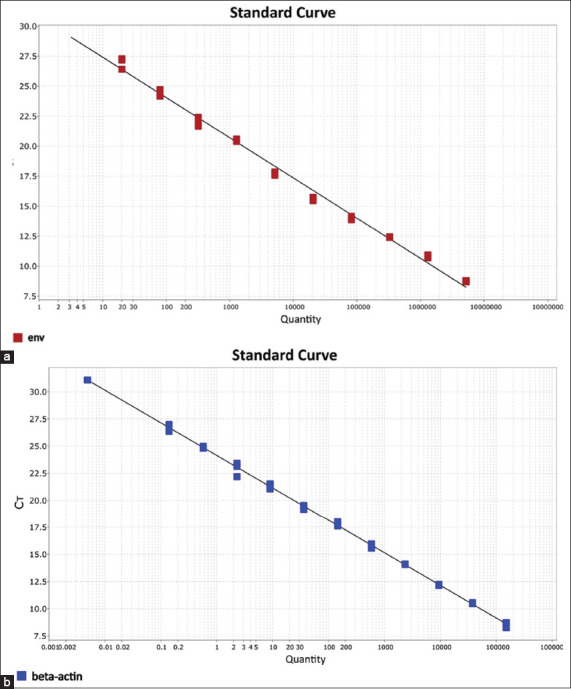
Standard curves based on real-time multiplex polymerase chain reaction results: (a) Detection of the bovine leukemia virus (BLV) env gene using a plasmid containing the cloned env gene; (b) Detection of the β-actin control gene in bovine genomic DNA samples.

qPCR assays using plasmid DNA for the *env* gene produced an R^2^ = 0.992, indicating high linearity. Ampli-fication of *β-actin* from serial dilutions of bovine DNA yielded an even higher R^2^ = 0.998. As illustrated in Figure 2, amplification efficiencies were 100.3% for the *env* gene and 115.5% for *β-actin*, with regression slopes ranging from −2.999 to −3.313, all within acceptable ranges for qPCR performance.

#### Specificity testing

**Figure 3:**
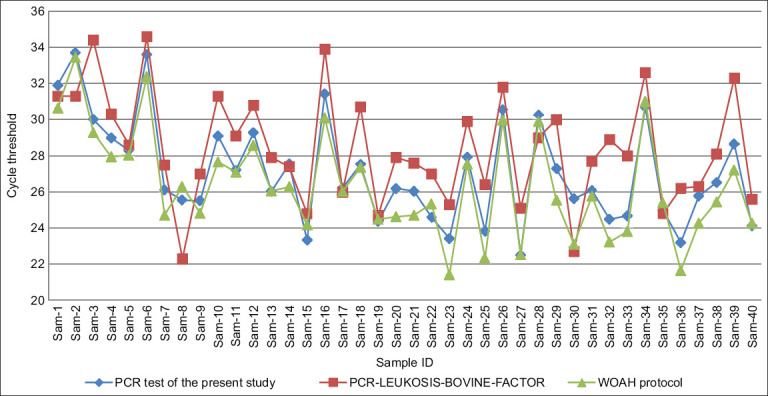
Comparison of threshold cycles for fluorescent signal detection using the developed polymerase chain reaction test, a commercial test system, and the World Organization for Animal Health protocol for detecting bovine leukemia virus. The graph shows the absolute cycle values, including the initial cycles that are not considered in the final calculation.

The assay’s specificity was evaluated using 62 DNA samples collected from a commercial dairy farm over a 5-year period with known BLV exposure. All three test systems–the developed assay, a WOAH-recommended protocol [16], and a commercial kit (“PCR-Leukemia-Cattle Factor,” VetFactor)—detected BLV in the same 40 samples. No fluorescent signals were observed in the remaining 22 samples with any method (Figure 3).

When comparing Ct values, the developed assay showed an average Ct delay of 0.72 cycles relative to the WOAH protocol (p < 0.001). The commercial kit demonstrated an even greater lag, with Ct values averaging 2.07 cycles later than the WOAH protocol (p < 0.001). These findings confirm strong concordance between the developed assay and the international reference protocol. The higher Ct values observed with the commercial kit may result from differences in DNA extraction protocols compared to the manufacturer’s specifications.

### Prevalence of BLV across regions

The developed qPCR assay was applied to 1,680 cattle samples collected from six regions of Kazakhstan: Akmola, Aktobe, Kostanay, Pavlodar, North Kazakhstan, and East Kazakhstan. BLV proviral DNA was detected in 654 animals, yielding an overall prevalence of 38.9%. Regional infection rates varied considerably, with East Kazakhstan showing the highest prevalence at 60.5%, followed by North Kazakhstan (50.3%), Kostanay (46%), Akmola (41%), Aktobe (17.4%), and Pavlodar with the lowest rate at 13% (Table 2).

**Table 2:** Prevalence of BLV in 6 regions of Kazakhstan and circulating genotypes.

Region	Positive No./Tested no. by qPCR	Percentage of positive animals	Number of sequences	Genotype (quantity)
Akmola	82/200	41	12	G4 (8) G7 (4)
Aktobe	33/190	17,4	6	G4 (6)
Kostanay	197/428	46	42	G8 (1) G4 (40) G1 (1)
Pavlodar	39/300	13	9	G4 (8) G7 (1)
North Kazakhstan	182/362	50,3	48	G4 (29) G7 (9) G1 (10)
East Kazakhstan	121/200	60,5	32	G7 (2) G8 (3) G4 (27)
Total	654/1680	38,9	149	G4 (118) G7 (16) G1 (11) G8 (4)

BLV=Bovine leukemia virus

### Genotype distribution and phylogenetic analysis

For genotyping, 149 qPCR-positive samples with Ct values below 28 were randomly selected. Of these, nes-ted PCR targeting a 594-bp *env* gene fragment yielded 122 usable sequences. An additional five sequences were obtained using 444-bp target primers, and the remaining 22 were successfully amplified using an alternative nested PCR approach.

**Figure 4:**
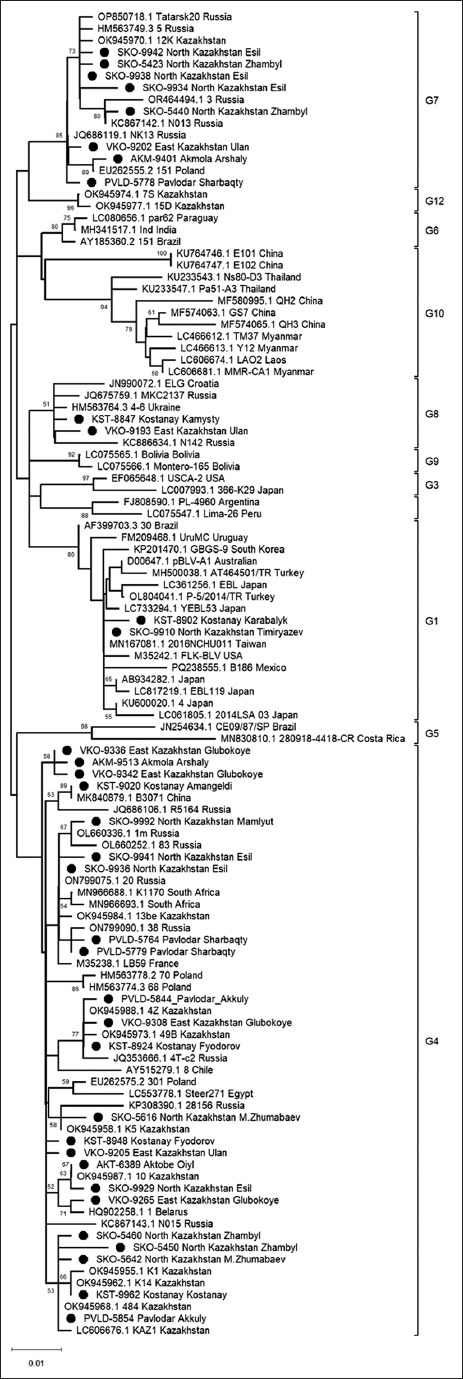
A phylogenetic tree showing the clustering of genotypes identified in this study with globally representative genotypes**.** The tree was constructed using the maximum likelihood method with the Kimura-2 model and gamma distribution (K2 + G), based on a 400-base pair fragment of the *bovine leukemia virus* gene. Twelve known reference genotypes are labeled as G1–G12. Local sequences identified in this study are marked with black circles and cluster within global genotypes based on their phylogenetic position relative to reference sequences.

Phylogenetic analysis of a 400-bp *env* gene region across all 149 sequences revealed 35 distinct gen-otypes. A representative sequence from each genotype was used for further phylogenetic analysis alongside 12 globally recognized BLV reference genotypes. As illustrated in Figure 4, genotype G4 was predominant, identified in 118 samples across all six regions (Table 2). G7 was detected in 16 samples from four regions, while G1 and G8 were identified in 11 and 4 samples, respectively, from Kostanay, North Kazakhstan, and East Kazakhstan.

To contextualize findings, a comprehensive review of BLV *env* gene sequences from the NCBI database (accessed December 2024) was conducted, focusing on sequences ≥400 bp. Table 3 summarizes the global distribution of 1862 such sequences.

**Table 3:** Geographic distribution and frequency of BLV *env* gene genotypes (400 bp fragment) based on 1862 sequences deposited in the NCBI database.

Genotype	Country (number of sequences)	Total	Percentage
G1	Argentina (2), Belarus (2), Belgian (13), Chile (5), China (3), Egypt (62), France (1), Germany (1), Japan (1), Kazakhstan (35), Moldova (2), Mongolia (6), Peru (5), Poland (6), Russia (348), South Africa (7), Ukraine (1), USA (2), Zambia (1)	503	27.0
G2	Argentina (17), Bolivia (1), Brazil (1), Paraguay (3), Peru (2)	24	1.3
G3	Colombia (3), Japan (16), Mexico (1), South Korea (2), and Taiwan (1)	23	1.2
G4	Argentina (11), Australia (1), Bhutan (1), Bolivia (3), Brazil (5), Colombia (142), Costa Rica (2), Dominica (2), Egypt (32), Iran (7), Japan (428), Japon (1), Mexico (45), Mongolia (5), Myanmar (4), Pakistan (20), Paraguay (6), Peru (34), Russia (1), Saint Kitts and Nevis (5), South Africa (1), South Korea (23), Taiwan (29), Thailand (13), Turkey (58), Uruguay (11), USA (2), Viet Nam (15), Zambia (14)	921	49.5
G5	Brazil (4), Costa Rica (4)	8	0.4
G6	Argentina (2), Bolivia (6), Brazil (12), China (29), Colombia (4), India (10), Myanmar (9), Pakistan (9), Paraguay (8), Peru (4), Thailand (8), and Vietnam (7)	108	5.8
G7	Australia (2), Brazil (1), Chile (3), Kazakhstan (7), Moldova (12), Mongolia (1), Poland (1), Russia (133), Ukraine (6), Italy (1)	167	9.0
G8	Croatia (6), Russia (8), Ukraine (4)	18	1.0
G9	Bolivia (22)	22	1.2
G10	China (11), Laos (2), Madagascar (1), Myanmar (21), Thailand (20), and Vietnam (7)	62	3.3
G11	China (2)	2	0.1
G12	Kazakhstan (4)	4	0.2

BLV=Bovine leukemia virus, NCBI=National Center for Biotechnology Information, bp=Base pair

Genotypes G1 and G4 were globally widespread, accounting for 503 and 921 sequences, respectively, together comprising 76.5% of all sequences. G1 was reported in 19 countries, with the highest frequency in Russia (348 sequences) and Egypt (62), suggesting dominance in Europe and Africa. G4 appeared in 29 countries, with particularly high numbers in Japan (428) and Colombia (142), indicating widespread distribution in Asia and South America.

Other genotypes showed restricted geographical patterns: G2, G5, G6, and G9 were mostly found in South America; G3, G6, G10, G11, and G12 were primarily Asian; and G7 and G8 were observed in Europe. Certain were country-specific – G9 in Bolivia (22 sequences), G11 in China (2), and G12 in Kazakhstan (4) – highlighting localized evolutionary lineages within specific regions.

## DISCUSSION

### Utility of molecular tools in the final stages of BLV eradication

Farm-level eradication of BLV typically relies on the identification and culling of infected animals using serological methods such as RID or ELISA. However, these tools are most effective during the mid-to-late stages of infection. In contrast, PCR and real-time PCR (qPCR) enable the detection of proviral DNA during early infection, before seroconversion occurs [34–37]. This early detection capacity is crucial in the final phases of eradication programs. Moreover, developing and validating in-house PCR assays offers a cost-effective alternative to commercial kits [38].

In this study, we developed a duplex qPCR assay that targets the *env* gene of BLV and incorporates bovine *β-actin* as an internal control. This assay provides a sensitive and efficient method for detecting BLV provirus, with validation against other diagnostic systems. The assay achieved a detection limit of 20 copies per reaction for the *env* gene and 6 copies for *β-actin*, aligning well with previously reported sen-sitivities for BLV qPCR ranging from 1 to 150 copies per reaction [39, 40].

### Specificity and comparative validation

The specificity of the developed assay was conf-irmed through both *in silico* and *in vitro* evaluations. Sequence alignment of 242 full-length BLV genomes enabled the design of primers targeting conserved regions of the *env* gene, with no mismatches at the 3′ ends. Degenerate bases were introduced to increase genotype coverage. In *in vitro* validation, the assay demonstrated full concordance with both the WOAH-recommended protocol and a commercial PCR kit in identifying positive and negative samples. The minor difference in Ct values between the developed and WOAH assays (0.72 cycles) further confirms the diagnostic relia-bility of the assay.

### Prevalence of BLV in Kazakhstan

Using the developed duplex qPCR assay, BLV was detected in 654 out of 1,680 DNA samples from cattle across six regions of Kazakhstan, yielding an overall prevalence of 38.9%. Considerable regional variation was observed: East Kazakhstan exhibited the highest prevalence at 60.5%, followed by North Kazakhstan (50.3%) and Kostanay (46.0%). The low-est prevalence was reported in Pavlodar (13.0%) and Aktobe (17.4%).

These findings are consistent with a prior study by Sultanov *et al*. [23], which reported an average infection rate of 23.7% using AGID and ELISA across 18 industrial dairy farms in six regions. However, the current study reveals greater regional variability, likely due to the inclusion of older animals and herds from private farms. Notably, our reported prevalence is substantially higher than the official national averages (3%) from 2002 to 2015 [41], suggesting an under-estimation in historical surveillance data.

### Global context and challenges in BLV control

EBL continues to pose a significant threat to cattle health and the global dairy industry. The WOAH mandates annual reporting of EBL status from its member states [16]. Control strategies differ signi-ficantly between countries, impacting the spread and persistence of BLV. Since the 1960s, nations in Western Europe, Scandinavia, and Oceania have implemented rigorous eradication programs based on widespread testing and culling, leading to EBL-free status in many cases [42, 43].

In contrast, several countries–including the United States, Brazil, China, and Argentina–continue to report high BLV prevalence, particularly in dairy cattle, where seropositivity can reach 50% [44–47]. In such settings, universal culling is often economically unfeasible. Consequently, alternative control strategies are being investigated, including the use of qPCR to quantify proviral load (PVL) and selectively remove high-shedding “super-spreaders” [45, 48–51].

### Genotypic diversity and transmission pathways

Molecular genotyping in this study identified four BLV genotypes circulating in Kazakhstan: G4 (79.1%), G7 (10.7%), G1 (7.4%), and G8 (2.7%). Genotype G4 was the most widely distributed, detected in all six regions. This genotype has also been reported in Russia, China [52–54], South America [55], and parts of Africa [56], confirming its global distribution. Genotype G1, another highly prevalent lineage, has been detected across all inhabited continents [57, 58].

The widespread presence of G1 and G4 supports the hypothesis that global cattle trade and breeding programs have facilitated their dissemination [59]. In contrast, G7 and G8 appear to have more localized distributions, primarily within Eastern Europe and Russia [60].

Kazakhstan exhibits considerable BLV genetic diversity, with five of the twelve globally recognized genotypes identified in its cattle population–four of which were confirmed in this study. This diversity likely stems from historical and modern importation of breeding animals, especially during the late 19^th^ and 20^th^ centuries when cattle were imported from Russia and across Europe to enhance local herds [61, 62].

## CONCLUSION

This study successfully developed and validated a duplex qPCR assay for the detection of BLV in cattle, targeting the conserved *env* gene and incorporating *β-actin* as an internal control. The assay demonstrated high analytical sensitivity–detecting as few as 20 copies per reaction for *env* and 6 GEs for *β-actin*–and strong specificity validated against both WOAH protocols and a commercial test kit. When applied to 1,680 bovine samples across six regions of Kazakhstan, the assay revealed a high overall BLV prevalence of 38.9%, with East Kazakhstan exhibiting the highest infection rate (60.5%) and Pavlodar the lowest (13%).

The genotyping component identified four circulating BLV genotypes (G1, G4, G7, and G8), with G4 being predominant (79.1%). These findings reflect significant molecular diversity within Kazakhstan’s BLV population, suggesting historical viral introductions through the importation of breeding stock. The study also contributes valuable molecular epidemiological data, revealing genotype overlaps with global BLV strains, notably G1 and G4, which are widely distributed across continents.

From a practical standpoint, the validated duplex qPCR assay offers a cost-effective, sensitive, and scalable diagnostic tool for early detection of BLV, including in young animals with maternal antibodies. Its use can enhance disease surveillance, guide culling strategies, and support targeted interventions, especially in regions with emerging or persistent outbreaks. The internal *β-actin* control ensures reliability by minimizing false negatives due to sample degradation or reaction failure.

The study’s strengths include a large-scale sample size, rigorous assay validation, the incorporation of an internal control, and comprehensive genotyping across multiple administrative regions. These colle-ctively ena-ble both accurate detection and in-depth molecular characterization of BLV in the Kazakh cattle population.

However, the study has limitations. Sampling was restricted to cattle over 3 years of age and excluded sou-thern regions of Kazakhstan, potentially underrepre-senting younger or geographically diverse populations. Furthermore, PVL quantification was not assessed, which could have provided insights into viral transmission dyn-amics and “super-spreader” identification.

Future research should expand to include natio-nwide sampling, longitudinal monitoring of infected herds, and integration of PVL quantification to inform selective culling policies. In addition, molecular surv-eillance should be regularly updated to detect any emerging BLV genotypes that may influence vaccine or diagnostic development.

In conclusion, this study provides a reliable mole-cular diagnostic tool and updated epidemiological data for BLV in Kazakhstan. The findings support the integ-ration of qPCR-based detection into national control and eradication programs and contribute to global eff-orts to mitigate the economic and health impacts of BLV in livestock.

## AUTHORS’ CONTRIBUTIONS

AO: PCR optimization, statistical analysis, sequ-encing, and drafted the manuscript. AS: Experimental design, data analysis, interpretation, and drafted and revised the manuscript. MK: Methodology, data collection, and drafted the manuscript. AD and DK: PCR, DNA extraction, visualization, and validation. AR and YA: Data interpretation and drafted the manuscript. KM: Project design and supervision. All authors have read and approved the final manuscript.
